# 

**Published:** 1889-06-01

**Authors:** 


					r Ho. 140, Vol. Vi-
[Registered at the General Post Office as a Newspaper.]
% MM Jg* m"ttoTe^nn^;V,. Vd" poVt iS.

				

